# Labour-type physical activity, metabolic dysregulation, and hypertension in rural older adults: rethinking work, exercise, and health in a cold-climate agricultural community

**DOI:** 10.3389/fpubh.2026.1761980

**Published:** 2026-03-06

**Authors:** Yan Gao, Ziyi Guo, Yongheng Zhao, Xuefeng Xi, Gaixia Hou, Limeng Liu, Dehui Zhang

**Affiliations:** 1 School of Physical Education, Jiamusi University, Jiamusi, China; 2 School of Journalism and Communication, Xiamen University, Xiamen, China; 3 School of Wushu, Henan University, Kaifeng, China; 4 Institute of Aging Studies, Henan University, Kaifeng, China; 5 School of Physical Education and Health Science, Mudanjiang Normal University, Mudanjiang, China; 6 Dongcheng Community Health Service Center, Wangkui, China

**Keywords:** ageing, hypertension, labour-type physical activity, leakage-free machine learning, metabolic dysregulation, rural health, structured exercise

## Abstract

**Background:**

Hypertension disproportionately affects older adults in rural settings. Labour-type physical activity is commonly assumed to protect cardiovascular health, yet it may not replicate the physiological stimulus of structured exercise. Evidence remains limited regarding how labour intensity and blood pressure-free obesity–metabolic phenotypes relate to hypertension in cold-climate rural populations.

**Methods:**

This community-based cross-sectional study analysed 2,191 adults aged ≥65 years from a government health examination programme in a cold-climate agricultural county in Northeast China. Blood pressure-free obesity–metabolic phenotypes were defined using BMI, triglycerides (TG), and HDL-C only. Physical activity was quantified using a cohort-specific PA Index (weekly frequency × duration) and categorised as inactive (0), low active (0–<180), and high active (≥180; median among active participants). We fitted multivariable logistic regression models for prevalent hypertension, excluding blood pressure (BP) variables to avoid circularity, and developed leakage-free prediction models using non-haemodynamic predictors.

**Results:**

The prevalence of hypertension was 75.9%. Hypertension prevalence differed across BP-free phenotypes (*χ^2^* = 28.07, *p* < 0.001), ranging from 75.1% (MHNO) to 89.7% (MUO). Compared with inactive participants, the odds of prevalent hypertension were higher in the low active group (OR = 1.85, 95% CI 1.32–2.58) and the high active group (OR = 2.00, 95% CI 1.61–2.49) (both *p* < 0.001). In leakage-free prediction, the primary LASSO model achieved a test-set ROC-AUC of 0.664 and PR-AUC of 0.841, with good calibration (slope 1.10).

**Conclusion:**

In this cold-climate rural cohort, BP-free metabolic–obesity phenotypes and higher labour-type activity volume were independently associated with higher odds of prevalent hypertension, which is not consistent with the assumption that “work equals exercise.” Leakage-free prediction using routinely collected screening variables may help inform outreach prioritisation and follow-up planning in resource-limited agricultural communities, pending external validation and implementation evaluation.

## 1 Introduction

Population ageing represents one of the most profound demographic transformations of the 21st century, reshaping the global burden of chronic disease and posing significant challenges to healthcare, social systems, and community well-being (1). Hypertension is widely recognised as a principal determinant of functional decline, disability, and premature mortality among older adults. Its impact is disproportionately severe in rural and resource-limited settings, where healthcare access is constrained, and prevention infrastructure remains fragile (2, 3). Maintaining effective blood pressure control is therefore a foundational prerequisite for sustaining mobility independence, autonomy, and quality of life in later life (4). In China, where a substantial proportion of the older population resides in rural agricultural communities, the prevalence and control rates of hypertension reveal widening rural–urban disparities, emphasising an urgent need for evidence-based strategies tailored to high-risk environmental and socioeconomic contexts (5). However, limited empirical evidence exists regarding how work patterns, ecological constraints, and cardiometabolic vulnerability converge to shape hypertension risk in ageing rural populations.

A long-standing assumption in public health and community practice is that agricultural labour—characterised by daily tasks such as crop cultivation, livestock handling, and heavy domestic work—naturally provides sufficient physical activity to maintain cardiovascular resilience (6). This belief equates physical fatigue with exercise-derived physiological benefits. However, emerging sports science and cardiovascular physiology research challenges this conventional logic, demonstrating that labour-type workloads typically involve repetitive mechanical loading, isometric exertion, and fluctuating low-to-moderate intensities lacking the sustained aerobic stimulus necessary for cardiorespiratory adaptation, vascular elasticity improvement, or autonomic balance enhancement (7, 8). This raises a critical conceptual question: Does being exhausted from work truly translate into the cardioprotective effects of structured exercise, or does labour fatigue disguise underlying physiological vulnerability? Empirical evidence addressing this “work–exercise paradox” in older rural populations remains extremely limited, leaving a critical gap in understanding how labour fatigue may conceal, rather than prevent, cardiovascular vulnerability (9).

Parallel to this paradigm shift in understanding physical activity, increasing attention has been directed toward metabolic phenotypes as more nuanced indicators of cardiometabolic vulnerability. Metabolic dysregulation—reflected by elevated triglycerides, low high-density lipoprotein cholesterol (HDL-C), and related lipid–insulin disturbances—has been identified as a strong determinant of hypertension risk independent of body size or total workload (10–12). The presence of phenotypes such as metabolically unhealthy non-obese individuals and metabolically healthy obese individuals demonstrates that obesity classification alone masks substantial heterogeneity in cardiovascular risk profiles (13, 14). However, the interaction between labour-type physical activity and metabolic phenotypes in shaping hypertension patterns in rural ageing populations remains poorly understood, limiting the ability to design precise intervention strategies (15, 16).

Cold-climate agricultural regions, such as those in Northeast China, provide a critically important but understudied context for examining these interrelations. Extreme winter temperatures restrict outdoor mobility and contribute to prolonged indoor confinement, reducing opportunities for structured aerobic activity (17). Seasonal dietary patterns characterised by high-salt intake, heating-related environmental stressors, and inflammatory responses associated with cold exposure may further exacerbate cardiovascular strain and increase the risk of hypertension (18–20). These ecological challenges intersect with structural health inequities, including limited primary-care resources, inadequate routine screening, and underdiagnosis, collectively widening geographic disparities in hypertension control (21). Understanding how labour-type physical activity and metabolic dysregulation interact within this environmental and systemic context is essential for realistic and regionally adapted prevention models.

Beyond clarifying epidemiological relationships, there is increasing recognition that innovative strategies are needed to improve early identification and risk stratification among older adults in resource-constrained settings (21). Traditional epidemiological approaches describe risk but provide limited utility for real-world decision-making. Artificial intelligence (AI) and machine-learning approaches offer promising tools for predictive screening and personalised intervention planning. In rural primary-care programmes, screening capacity may be intermittent and staff-limited; therefore, pragmatic triage tools are needed to prioritise outreach, repeat measurements, and follow-up among individuals most likely to be hypertensive. However, methodological rigour is essential: models must avoid performance inflation caused by the inclusion of outcome-defining variables, such as directly measured blood pressure. This challenge is addressed by leakage-free modelling frameworks designed to simulate real-world pre-measurement screening conditions (22), in which risk stratification is performed using routinely collected demographic, anthropometric, biochemical, and lifestyle information before standardised blood pressure assessment is feasible. Moreover, integrating potentially discordant signals—such as high labour-type activity volume alongside adverse metabolic profiles—may improve risk stratification in populations where “work” does not equate to cardioprotective exercise. When implemented responsibly, explainable AI has the potential to support primary-care decision-making, enhance resource allocation, and improve hypertension detection in rural settings with limited clinical capacity (23, 24), acting as a decision-support adjunct that assists rather than replaces formal blood pressure measurement.

Against this background, the present study aims to: (1) characterise labour-type physical activity patterns and obesity–metabolic phenotypes among rural older adults in a cold-climate agricultural community; (2) examine associations between metabolic dysregulation and hypertension within a population dominated by low-to-moderate labour activity; and (3) evaluate AI-enabled, leakage-free machine-learning models for hypertension prediction using routinely collected demographic, anthropometric, biochemical, and lifestyle data to support pre-measurement triage and prioritised follow-up when screening resources are limited. By re-examining the relationships among work, exercise, and cardiovascular health, this study aims to provide an evidence-based foundation for targeted screening, structured exercise prescription, and community-based precision prevention strategies that contribute to healthy ageing and the reduction of rural–urban health inequities (25).

## 2 Methods

### 2.1 Study design and population

This community-based, cross-sectional study adhered to the Strengthening the Reporting of Observational Studies in Epidemiology (STROBE) guidelines for observational research (26). Data were derived from a government-organised health examination programme for community-dwelling older adults in a cold-climate agricultural county in Northeast China. The programme offers free annual screenings for residents aged 65 years and above, including standardised questionnaires, physical examinations, and fasting blood sampling, performed at township health centres or village clinics.

All examinations were conducted by uniformly trained physicians and nurses using national technical specifications for chronic disease surveillance (27). Laboratory tests were performed in a county-level central clinical laboratory that operates under both internal and external quality control procedures. The anonymised dataset was released to the research team under a data-use agreement with the local Health Bureau. The study protocol complied with the Declaration of Helsinki and was approved by the Institutional Ethics Committee of Henan University (Approval No. HUSOM2025-929) and the local Health Bureau. All participants had provided written informed consent at the time of the health examination.

**Figure 1:**
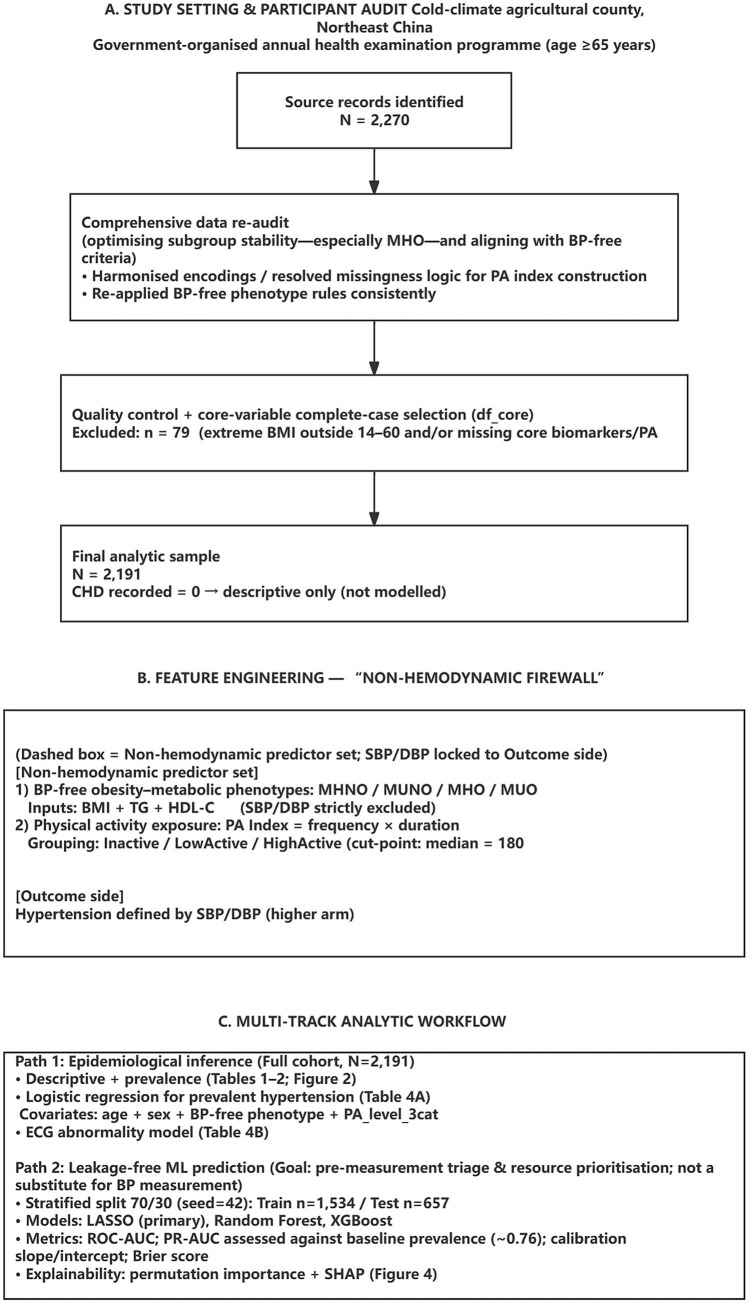
Study setting, participant flow, and analytic workflow for a community-based health examination in a cold-climate rural county in Northeast China.

This analysis focuses on the relationship between obesity phenotypes, physical activity, and cardiovascular outcomes (hypertension and electrocardiogram [ECG] abnormalities). A unified analytic dataset was constructed from a total of 2,270 participants, restricted to variables required for all subsequent analyses. Quality control filters and complete-case selection (see Section 2.4) yielded a final core analytic sample of 2,191 older adults. This expanded sample includes additional participants retrieved during a comprehensive data re-audit who met the refined blood pressure-free phenotype criteria. An overview of the study setting, participant flow, and analytic workflow is illustrated in Figure 1.

### 2.2 Variables and measurements

#### 2.2.1 Demographic characteristics

Age (years) and sex (male/female) were obtained from official registration and verified during face-to-face interviews by trained staff. Age was treated as a continuous variable in all models. Sex was coded as a binary indicator (0 = female, 1 = male).

#### 2.2.2 Anthropometric and biochemical measurements

Body weight and height were measured using calibrated scales and stadiometers with participants wearing light clothing and no shoes. Body mass index (BMI) was calculated as weight (in kilograms) divided by height squared (in metres squared). For quality control, implausible BMI values were excluded by restricting the analysis to the range of 14–60 kg/m^2^.

After an overnight fast of at least 8 h, venous blood samples were collected, transported under cold-chain conditions, and centrifuged within 2 h of collection. Serum triglycerides (TG) and high-density lipoprotein cholesterol (HDL-C) were measured using enzymatic methods on automated analysers, following the manufacturer’s protocols and national quality control standards (27). All assays participated in provincial external quality-assessment schemes.

#### 2.2.3 Blood pressure and cardiovascular outcomes

Blood pressure was measured using calibrated automated sphygmomanometers (e.g., Omron HBP-1300 or equivalent) after at least 5 min of seated rest. The cuff size was selected according to arm circumference. Blood pressure was measured in both arms, and the higher-arm values for systolic blood pressure (SBP) and diastolic blood pressure (DBP) were used in this analysis, consistent with population-based screening and measurement recommendations (28).

Three binary cardiovascular outcomes were derived from the health-examination records:

- Hypertension (primary outcome): defined as SBP ≥ 140 mmHg and/or DBP ≥ 90 mmHg using the higher-arm measurement, consistent with Chinese and international guideline thresholds for epidemiological studies (26).
- ECG abnormality: coded as present/absent based on standardised 12-lead resting ECG interpretation by trained clinicians.
- Coronary heart disease (CHD): recorded as present/absent only when *a prior* physician-diagnosed CHD history was documented in the health-examination form (i.e., recorded diagnosis/history rather than adjudicated incident events).

For analysis, hypertension, ECG abnormality, and CHD were each harmonised to binary variables (0 = no, 1 = yes) to ensure consistent encoding across factor/character formats in the original dataset. In the final analytic sample, no documented CHD history was recorded; therefore, CHD was reported descriptively only and not modelled.

#### 2.2.4 Obesity–metabolic phenotypes (blood pressure-free definition)

Obesity phenotypes were defined by combining BMI-based obesity status with metabolic abnormalities. To address methodological concerns regarding circular reasoning (tautology) when hypertension was the primary outcome, systolic and diastolic blood pressure components were strictly excluded from the metabolic abnormality criteria *a priori*. For each participant, the following criteria were applied: (i) Obesity: BMI ≥ 28.0 kg/m^2^ (Chinese adult obesity threshold); (ii) High triglycerides: TG ≥ 1.7 mmol/L; (iii) Low HDL-C: HDL-C < 1.0 mmol/L.

The number of metabolic abnormalities (high TG and/or low HDL-C) was summed (range 0–2), and four mutually exclusive phenotypes were defined: (1) Metabolically healthy non-obesity (MHNO): BMI < 28 kg/m^2^ and no abnormalities (sum = 0); (2) Metabolically unhealthy non-obesity (MUNO): BMI < 28 kg/m^2^ with ≥ 1 abnormality; (3) Metabolically healthy obesity (MHO): BMI ≥ 28 kg/m^2^ and no abnormalities; and (4) Metabolically unhealthy obesity (MUO): BMI ≥ 28 kg/m^2^ with ≥ 1 abnormality. These categories were implemented as a factor variable with the fixed level order: MHNO, MUNO, MHO, MUO. This BP-free definition ensures that associations with hypertension are not mechanically induced by diagnostic overlap. With the expanded analytic sample (*N* = 2,191), the MHO phenotype achieved a robust cell size (*n* = 134), allowing for reliable independent analysis of all four groups. Sensitivity analyses using collapsed categories (Obese vs. Non-obese) were additionally performed to confirm the consistency of the findings.

#### 2.2.5 Physical activity (cohort-specific grouping)

Physical activity was assessed using a standardised question on the weekly frequency and duration of sessions lasting at least 30 min, including walking, agricultural labour, and domestic work. A weekly activity volume index (PA Index) was derived as the product of frequency (sessions/week) and duration (min/session). To avoid misclassifying true inactivity as missingness, participants with an exercise frequency of 0 and missing duration were assigned a duration of 0 (i.e., PA Index = 0).

Given that guideline-based “high activity” thresholds (e.g., ≥5 sessions/week and ≥ 60 min/session) yielded empty categories in this rural cohort, we implemented a data-driven, cohort-specific grouping approach to ensure sufficient contrast for analysis. Participants were categorised into three levels: (1) Inactive: PA Index = 0; (2) Low active: 0 < PA Index < the median PA Index among active participants in the analytic sample (median = 180); and (3) High active: PA Index ≥180. To test robustness, sensitivity analyses subdivided active participants into tertiles (Active_T1, Active_T2, Active_T3) using the R function ntile(), which is robust to ties and prevents empty bins. This approach ensures that comparisons reflect the actual distribution of labour-type activity in this cold-climate agricultural setting rather than arbitrary external thresholds. Physical-activity type (exercise_type) was cleaned using a text normalisation procedure and treated as a categorical covariate only in exploratory machine-learning models.

#### 2.2.6 Lifestyle and other covariates

Two additional lifestyle indicators were extracted from the health-examination questionnaires:

- Diet type (diet_type): categorised according to self-reported typical diet pattern (e.g., light, moderate, salty) and cleaned using the same text-normalisation procedure as exercise_type.
- Smoking status (smoke_status): categorised as current, former, or never smoker, and similarly cleaned.

For the primary multivariable regression model of prevalent hypertension, we adjusted for age, sex, BP-free obesity–metabolic phenotype (MHNO/MUNO/MHO/MUO), and the cohort-specific physical activity level (PA_level_3cat). Because the phenotype was constructed from BMI, triglycerides, and HDL-C, these components were not additionally entered to avoid redundancy and collinearity; SBP and DBP were excluded because they define the outcome.

For the ECG-abnormality outcome, SBP and DBP were retained as continuous clinical covariates.

Lifestyle factors (exercise type, diet type, smoking status) were included only in extended/sensitivity or exploratory machine-learning models to leverage pre-outcome information, but were not required for the primary regression or primary leakage-free prediction models. For the primary hypertension regression model, predictors were age, sex, BP-free obesity–metabolic phenotype (MHNO/MUNO/MHO/MUO; constructed from BMI and TG/HDL-C only), and the cohort-specific PA level (inactive/low active/high active). To avoid redundancy and collinearity, BMI, TG, and HDL-C were not entered as separate covariates in the primary phenotype-based model because they are embedded in the phenotype definition.

### 2.3 Data preparation and quality control

All analyses were performed using R (version 4.5.0; R Foundation for Statistical Computing, Vienna, Austria). Data management and descriptive statistics were conducted using the dplyr and tidyr packages, while visualisation was performed with ggplot2. Classical regression was implemented using base R and the stats package, and machine-learning modelling was carried out with glmnet, randomForest, caret, xgboost, pROC, and PRROC. The data-preparation steps followed the analytic workflow summarised in Figure 1.

A core analytic dataset (df_core) was constructed by:

1. Importing the raw de-identified dataset (clean.csv).
2. Retaining only variables required for the pre-specified primary analyses (phenotypes, regression, and leakage-free ML): outcomes (hypertension, ECG abnormality, CHD), demographics (age, sex), anthropometrics and lipids (BMI, TG, HDL-C), physical activity (exercise frequency, exercise duration; PA Index and PA_level_3cat derived), and (where applicable) SBP/DBP for descriptive purposes and ECG-outcome adjustment only.
3. Applying a physiological plausibility filter for BMI (14–60 kg/m^2^).
4. Performing complete-case selection for the above core variables, yielding the final analytic sample of 2,191 participants.

Binary outcomes were harmonised to 0/1 encoding, robust to initial storage as numeric, factor, or character. Text variables (exercise type, diet type, and smoking status) were cleaned via text normalisation and used only in exploratory supplementary models; they were not required for the primary regression or the primary leakage-free ML analyses (26).

### 2.4 Statistical analysis

#### 2.4.1 Descriptive statistics and prevalence comparisons

Baseline characteristics were summarised by obesity phenotype and three-level physical activity category (PA_level_3cat). For each phenotype-PA stratum, we reported the number of participants, mean age, mean BMI, and percentage of women (Table 1).

**Table 1:** Baseline characteristics by blood pressure-free obesity–metabolic phenotypes and cohort-specific physical activity levels (*n* = 2,191).

Panel A. By blood pressure-free obesity–metabolic phenotype								
Phenotype	*n*	Age (years), mean ± SD	Female, %	BMI (kg/m^2^), mean ± SD	TG (mmol/L), median [IQR]	HDL-C (mmol/L), mean ± SD	PA Index, median [IQR]	Hypertension, %
MHNO	1,003	73.1 ± 6.2	46.8	23.4 ± 2.6	1 [1, 1]	1.5 ± 0.3	90 [0, 180]	75.1
MUNO	889	72.7 ± 6.0	56.0	23.9 ± 2.5	2 [2, 3]	1.2 ± 0.3	90 [0, 180]	72.9
MHO	134	71.8 ± 5.3	46.3	30.1 ± 2.4	1 [1, 2]	1.5 ± 0.3	180 [0, 180]	85.1
MUO	165	71.4 ± 4.8	54.5	30.2 ± 1.9	2 [2, 3]	1.2 ± 0.2	180 [0, 180]	89.7

Panel B. By cohort-specific physical activity level (PA_level)							
PA_level	*n*	Age (years), mean ± SD	Female, %	BMI (kg/m^2^), mean ± SD	TG (mmol/L), median [IQR]	HDL-C (mmol/L), mean ± SD	Hypertension, %
Inactive	845	74.5 ± 6.9	50.8	23.7 ± 3.5	2 [1, 2]	1.4 ± 0.4	67.3
Low active	272	72.3 ± 5.4	52.2	24.8 ± 3.3	2 [1, 2]	1.4 ± 0.3	80.1
High active	1,074	71.4 ± 5.0	51.0	25.0 ± 3.1	2 [1, 2]	1.4 ± 0.3	81.7

Panel C. Cross-tabulation of phenotype × PA_level (cell counts)			
Phenotype\PA_level	Inactive	Low active	High active
MHNO	386	132	485
MUNO	374	100	415
MHO	41	17	76
MUO	44	23	98

Blood pressure-free obesity–metabolic phenotypes were defined using BMI and lipid profiles only (TG and HDL-C), with blood pressure strictly excluded to avoid outcome overlap: MHNO, BMI <28 kg/m^2^ and no lipid abnormality; MUNO, BMI <28 kg/m^2^ with ≥ 1 lipid abnormality; MHO, BMI ≥28 kg/m^2^ and no lipid abnormality; MUO, BMI ≥28 kg/m^2^ with ≥1 lipid abnormality. Lipid abnormalities were defined as TG ≥1.7 mmol/L and/or HDL-C < 1.0 mmol/L.

A weekly activity volume index (PA Index) was calculated as weekly frequency (sessions/week) × duration (min/session). PA_level was defined using cohort-specific cut-points: inactive (PA Index = 0), low active (0 < PA Index <180), and high active (PA Index ≥180), where 180 corresponds to the median PA Index among active participants.

Continuous variables are presented as mean ± SD or median [IQR] as indicated. Categorical variables are presented as n (%) unless otherwise specified. Hypertension was defined as SBP ≥ 140 mmHg and/or DBP ≥ 90 mmHg (higher arm measurement).

BMI, body mass index; TG, triglycerides; HDL-C, high-density lipoprotein cholesterol; IQR, interquartile range; PA, physical activity; SBP, systolic blood pressure; DBP, diastolic blood pressure.

For prevalence comparisons of cardiovascular outcomes, we focused on the full analytic sample and distinguished between the three binary outcomes defined in Section 2.2.3: hypertension, ECG abnormality, and CHD. Because no CHD events were recorded in the analytic dataset, CHD was described descriptively only and not modelled in regression or machine-learning analyses. This likely reflects underdiagnosis and/or recording limitations in routine village-level health examination forms (e.g., reliance on previously documented physician diagnoses rather than systematic case adjudication), rather than a true absence of CHD in this population.

**Figure 2:**
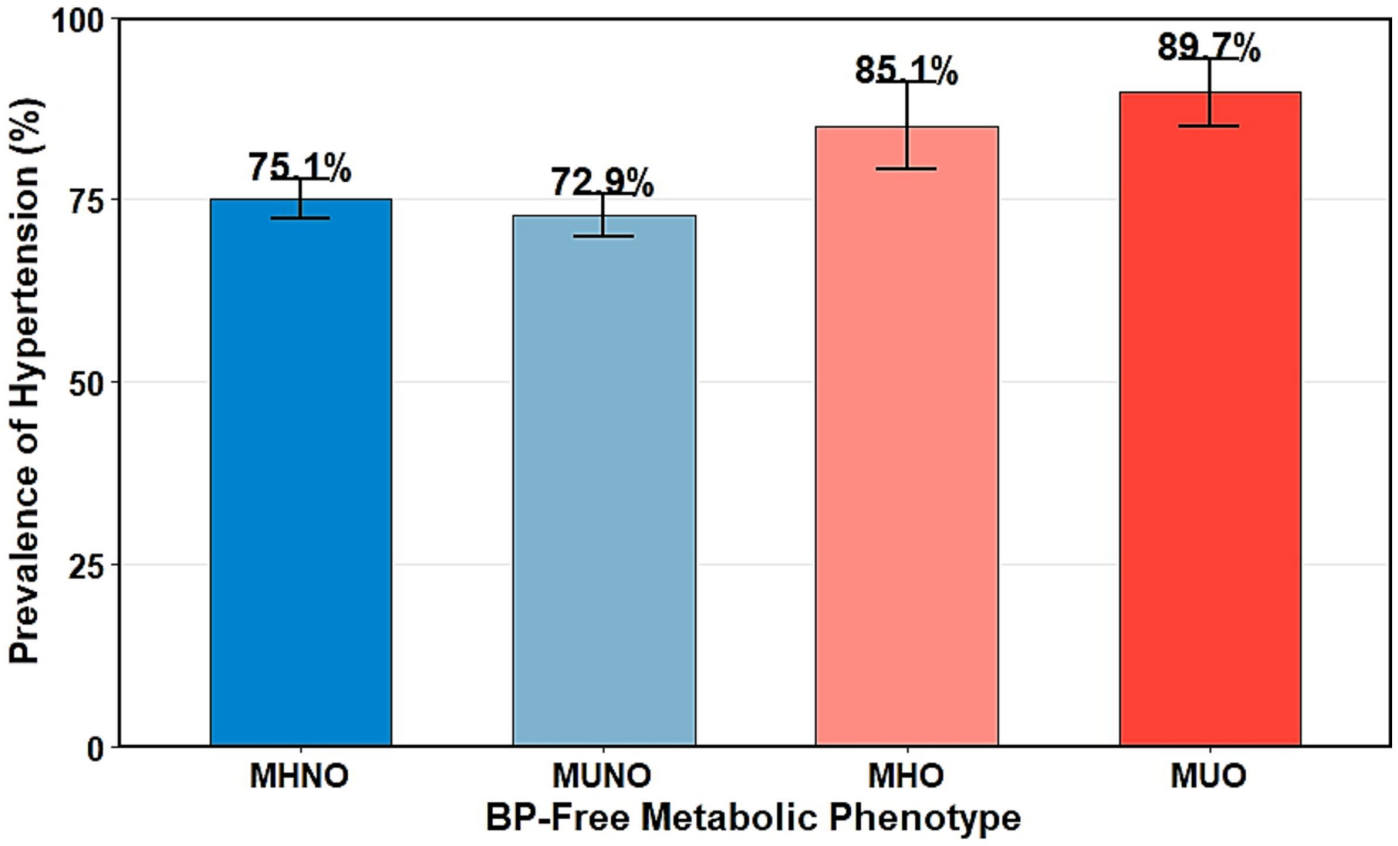
Prevalence of prevalent hypertension across blood pressure-free obesity–metabolic phenotypes. Bars show the prevalence of hypertension with 95% confidence intervals for MHNO (75.1%), MUNO (72.9%), MHO (85.1%), and MUO (89.7%). Phenotypes were defined using BMI and lipid markers (triglycerides and HDL-C) only, with SBP/DBP strictly excluded to avoid outcome overlap. Hypertension was defined using the higher-arm measurement as SBP ≥ 140 mmHg and/or DBP ≥ 90 mmHg. MHNO, metabolically healthy non-obese; MUNO, metabolically unhealthy non-obese; MHO, metabolically healthy obese; MUO, metabolically unhealthy obese; BMI, body mass index; HDL-C, high-density lipoprotein cholesterol; SBP, systolic blood pressure; DBP, diastolic blood pressure.

Prevalence of hypertension and ECG abnormalities was calculated across blood pressure-free obesity–metabolic phenotypes and across the cohort-specific three-level physical activity grouping (PA_level_3cat: inactive, low active, high active). Pearson’s *χ^2^* tests were used to assess overall heterogeneity across phenotypes for each outcome (Table 2; Figure 2).

**Table 2:** Prevalence of hypertension and ECG abnormalities across blood pressure-free obesity–metabolic phenotypes (*n* = 2,191).

Obesity–metabolic phenotype	*n*	Hypertension, *n* (%)	ECG abnormality, *n* (%)	CHD, *n* (%)
MHNO	1,003	753 (75.1)	563 (56.1)	0 (0.0)
MUNO	889	648 (72.9)	486 (54.7)	0 (0.0)
MHO	134	114 (85.1)	80 (59.7)	0 (0.0)
MUO	165	148 (89.7)	107 (64.8)	0 (0.0)

Blood pressure-free obesity–metabolic phenotypes were defined using BMI and lipid profiles only (TG and HDL-C), with blood pressure strictly excluded to avoid outcome overlap. Hypertension was defined as SBP ≥ 140 mmHg and/or DBP ≥90 mmHg (higher-arm measurement). Hypertension across phenotypes: Pearson’s χ^2^ = 28.073, df = 3, *p* = 3.506 × 10^−6^. ECG abnormality across phenotypes: Pearson’s χ^2^ = 6.497, df = 3, *p* = 0.0898. CHD was coded from documented physician-diagnosed history in routine examination records; no documented CHD history was recorded in this analytic dataset.

As a sensitivity analysis (not driven by sparse cells), obesity phenotypes were additionally collapsed into two categories—Non-obese (MHNO + MUNO) and Obese (MHO + MUO)—and analysed using 2 × 2 contingency tables to obtain odds ratios (ORs) and 95% confidence intervals (CIs) for hypertension and ECG abnormality (Table 3).

**Table 3:** Association between collapsed obesity phenotype and cardiovascular outcomes among older adults.

Outcome	Comparison (exposure)	Odds ratio (95% CI)	*p*-value
Hypertension	Obese vs. Non-obese	1.77 (1.21–2.58)	0.001
ECG abnormality	Obese vs. Non-obese	1.05 (0.81–1.37)	0.76

“Obese” includes MHO and MUO (*n* = 299); “Non-obese” includes MHNO and MUNO (*n* = 1892). Results are based on 2 × 2 contingency tables and Fisher’s exact tests. Obesity phenotypes were defined, excluding blood pressure to avoid circular reasoning.

Physical activity was analysed using the cohort-specific PA Index and its three-level grouping (inactive, low active, high active; Section 2.2.5). Guideline-based “high activity” thresholds (e.g., ≥5 sessions/week and ≥60 min/session) yielded empty categories in this rural cohort and were therefore not used for inference. Prevalence comparisons and regression models primarily used the PA_level_3cat grouping to reflect the observed distribution of labour-type activity exposure in this setting.

#### 2.4.2 Multivariable logistic regression

To examine independent associations between exposures and cardiovascular outcomes, we fitted multivariable logistic regression models for two binary outcomes: hypertension and ECG abnormality. To avoid circular reasoning and ensure outcome-appropriate adjustment, predictor sets were specified separately for each outcome.

Hypertension model (primary, phenotype-based). The primary model for prevalent hypertension included age (per 1-year increase), sex (female vs. male), blood pressure-free obesity–metabolic phenotype (MHNO as the reference category), and cohort-specific physical activity level (PA_level_3cat: inactive as reference; low active and high active). Because the BP-free phenotype was constructed from BMI and lipid markers (TG and HDL-C), BMI, TG, and HDL-C were not additionally entered as separate covariates in this primary phenotype-based model to avoid redundancy and collinearity. Systolic and diastolic blood pressure (SBP/DBP) were intentionally excluded from all hypertension models because they define the outcome.

ECG-abnormality model. The ECG-abnormality model included age, sex, obesity status (Obese vs. Non-obese), and physical activity status (active vs. inactive), consistent with Table 4B.

**Table 4:** Multivariable logistic regression for hypertension and ECG abnormalities (*n* = 2,191).

(A) Hypertension (BP-excluded model)		
Predictor	Odds ratio (95% CI)	*p*-value
Age (per 1-year increase)	0.98 (0.97–1.00)	0.048
Female sex (vs male)	1.12 (0.92–1.38)	0.240
Obesity phenotype (Ref: MHNO)		
MUNO	0.91 (0.74–1.13)	0.400
MHO (BP-free)	1.77 (1.07–2.92)	0.026
MUO (BP-free)	2.65 (1.56–4.49)	<0.001
PA level (Ref: inactive)		
Low active	1.85 (1.32–2.58)	<0.001
High active (PA Index ≥ 180)	2.00 (1.61–2.49)	<0.001

(B) ECG abnormalities		
Predictor	Odds ratio (95% CI)	*p*-value
Age (per 1-year increase)	1.03 (1.01–1.05)	0.009
Obesity (obese vs non-obese)		
Obese	1.05 (0.81–1.37)	0.760
PA level (active vs inactive)		
Active	1.15 (0.95–1.40)	0.150

BP-free obesity–metabolic phenotypes were defined using BMI and lipid markers (TG and HDL-C) only; SBP and DBP were not used in phenotype construction to avoid outcome overlap when modelling hypertension.

Model A (Hypertension) included age, sex, BP-free phenotype (MHNO as reference), and cohort-specific PA level (inactive as reference); SBP/DBP were not included as covariates.

Model B (ECG abnormality) included age, sex, obesity status (Obese vs Non-obese), and PA level (active vs inactive).

MHNO, metabolically healthy non-obese; MUNO, metabolically unhealthy non-obese; MHO, metabolically healthy obese; MUO, metabolically unhealthy obese; TG, triglycerides; HDL-C, high-density lipoprotein cholesterol; PA, physical activity; OR, odds ratio; CI, confidence interval; ECG, electrocardiogram; SBP, systolic blood pressure; DBP, diastolic blood pressure.

As robustness checks focused on exposure specification, sensitivity models evaluated physical activity using continuous exercise frequency and duration (instead of PA_level_3cat) while retaining the same core covariate structure. This outcome-specific predictor specification and exclusion of outcome-defining variables align with recommended epidemiological practice to minimise circularity and information leakage in observational modelling (26, 29).

All models were fitted using the glm function with the binomial family and logit link. Results are reported as odds ratios (ORs) with 95% confidence intervals (CIs) and two-sided *p*-values (Table 4).

#### 2.4.3 Machine-learning models for hypertension prediction

To complement classical regression and explore the joint predictive value of obesity phenotypes, physical activity, and clinical factors for hypertension, we implemented several supervised machine-learning models with hypertension as the outcome:

- Random forest.
- LASSO logistic regression (primary model).
- Extreme gradient boosting (XGBoost).

All machine-learning analyses were restricted to the same complete-case dataset (df_core), ensuring direct comparability with regression analyses. Given the absence of CHD events and the high prevalence but limited between-phenotype variation for ECG abnormalities, machine-learning models were not developed for ECG abnormalities or CHD.

##### 2.4.3.1 Feature construction

A common feature set was created using model.matrix() (dummy-coding categorical variables). To ensure a strict leakage-free framework, all primary machine-learning models were specified *a priori* to exclude systolic and diastolic blood pressure (SBP/DBP) from the predictor set. To avoid redundancy and potential collinearity, we pre-specified a component-based primary feature set and treated the phenotype representation as a sensitivity specification (see below). The resulting non-haemodynamic feature set included:

- Age and sex.
- Anthropometric and lipid components: BMI, triglycerides, and HDL-C (continuous).
- Physical-activity exposure captured by PA Index and the cohort-specific three-level PA grouping (inactive, low active, high active; derived from weekly frequency × duration), with inactive (PA Index = 0) explicitly retained as a separate category.
- BP-free obesity–metabolic phenotype (MHNO, MUNO, MHO, MUO) was evaluated as an alternative (sensitivity) feature representation replacing BMI/TG/HDL-C, rather than being entered simultaneously with its components, to prevent “double counting” embedded information.
- Lifestyle indicators (diet type, smoking status, and exercise type; dummy-coded) were included only when available and were treated as exploratory predictors.

For transparency, SBP/DBP–included models (when reported) were treated as supplementary, non-leakage-free comparisons and were not used as the primary evidence for clinical utility. Continuous exercise frequency and duration were retained only in sensitivity feature sets to represent physical-activity volume where applicable, but the PA Index framework was prioritised to reflect labour-type activity distribution in this cohort. Accordingly, primary ML performance results reported in the main text are based on the leakage-free, component-based predictor set (age, sex, BMI, TG, HDL-C, and PA metrics), while phenotype-based ML results (if reported) are presented as sensitivity analyses to assess robustness to feature representation.

##### 2.4.3.2 Data splitting and internal validation

All machine-learning models used the same stratified 70/30 train–test split to preserve hypertension prevalence and ensure comparability across algorithms. With a fixed random seed of 42, 2,191 participants were split into a training set (70%) and a held-out test set (30%, *n* = 657).

Within the training set, the LASSO model underwent 5-fold cross-validation to select the optimal regularisation parameter (lambda.min) that minimised binomial deviance. No external validation cohort was available; therefore, model performance was evaluated internally on the held-out test set, providing a conservative estimate of out-of-sample discrimination (30).

##### 2.4.3.3 Model specifications and performance metrics

Performance metrics were pre-specified to reflect both discrimination and clinical usability in a high-prevalence cohort. Discrimination was assessed by ROC-AUC and Precision–Recall AUC (PR-AUC; Average Precision). Classification metrics included accuracy, sensitivity, specificity, precision, and F1-score. Calibration was evaluated using the Brier score, calibration-in-the-large (intercept), and calibration slope. Results are summarised in Table 5 (and decision thresholds in Table 6, where applicable). PR-AUC (Average Precision) was computed from test-set predicted probabilities using a precision–recall implementation (e.g., PRROC), consistent across algorithms.

**Table 5:** Predictive performance of the leakage-free LASSO model (*n* = 2,191).

Metric	Value	Note
AUC (ROC)	0.664	Discrimination ability between hypertensive and non-hypertensive individuals.
PR-AUC (AP)	0.841	Area under the Precision–Recall curve; more informative for high-prevalence cohorts.
Brier Score	0.173	Measures the mean squared difference between predicted probability and actual outcome.
Calibration Slope	1.10	Ideal value is 1.0; it reflects the agreement between predicted and observed risks.
Calibration Intercept	−0.17	Reflects the distance between the average predicted and observed risks.

Leakage-free Framework: All models were trained and tested, excluding systolic and diastolic blood pressure from the input features to ensure independent predictive assessment and avoid circular reasoning.

Data Split: Results are reported on an independent held-out test set (*n* = 657), representing 30% of the total analytic sample.

AUC, area under the receiver operating characteristic curve; PR-AUC, precision–recall area under the curve; AP, average precision; ROC, receiver operating.

Characteristic: The primary model shown is the LASSO (Least Absolute Shrinkage and Selection Operator) logistic regression, selected for its optimal balance of parsimony and predictive performance.

**Table 6:** Clinical decision thresholds for hypertension screening.

Rule	Threshold	Accuracy	Sensitivity	Specificity
Balanced (Youden)	0.76	0.65	66.0%	62.5%
Screening (Se 90%)	0.66	0.75	90.1%	29.4%

“Leakage-free” indicates that SBP and DBP were excluded from predictors. Results are based on the independent test set (*n* = 657).

###### 2.4.3.3.1 Random forest

A random-forest classifier (randomForest package) was trained using default tuning with ntree = 100. Test-set predicted probabilities were used to derive ROC-AUC and PR-AUC, with classification metrics computed at the selected decision threshold(s) (Table 5).

###### 2.4.3.3.2 LASSO logistic regression (primary model)

The primary model was a leakage-free penalised logistic regression with L1 regularisation (glmnet; alpha = 1) fitted using the non-haemodynamic feature set (excluding SBP/DBP). The optimal penalty parameter was selected via 5-fold cross-validation (lambda.min). The final model generated test-set predicted probabilities, from which ROC-AUC, PR-AUC, and classification metrics were calculated (Table 5). Calibration was assessed using the Brier score, calibration intercept, and calibration slope estimated by regressing observed outcomes on the logit of predicted probabilities in a secondary logistic model. Non-zero coefficients were extracted for interpretability.

To facilitate real-world implementation, decision thresholds were evaluated using (i) the Youden-index-derived threshold and (ii) a sensitivity-priority threshold targeting ~90% sensitivity, enabling triage-oriented screening (Table 6).

###### 2.4.3.3.3 XGBoost

Gradient-boosted decision trees were trained using the xgboost package with: objective = “binary:logistic,” eval_metric = “auc” (training monitoring), learning rate eta = 0.03, max_depth = 3, subsample = 0.8, colsample_bytree = 0.8, and 300 boosting rounds. The model was trained on the training set DMatrix and evaluated on the held-out test set to obtain ROC-AUC and PR-AUC computed *post hoc* from predicted probabilities, with secondary classification metrics summarised in Table 5. XGBoost was used primarily as an explainability vehicle (permutation importance and SHAP) under the leakage-free specification.

##### 2.4.3.4 Feature importance and explainability

To interpret model behaviour under the leakage-free framework, explainability analyses were conducted using the leakage-free XGBoost model trained without SBP/DBP.

- Permutation importance: For each feature in the test set, values were permuted in turn, and the resulting decrease in AUC relative to the baseline test AUC was computed. Features were ranked by the magnitude of AUC drop, with the top contributors presented in Figure 4A.
- SHAP summary analysis: SHAP values were obtained from the trained leakage-free XGBoost model (predcontrib = TRUE), excluding the bias term. For each feature, the mean absolute SHAP value quantified its average contribution to the log-odds of hypertension across test observations. A SHAP summary plot was generated for the leading predictors (Figure 4B). This approach provides a transparent explanation of how non-haemodynamic predictors (e.g., BMI, labour-type activity volume captured by PA Index/high active status, and lipid markers) drive predicted hypertension risk under the leakage-free framework, avoiding circularity introduced by SBP/DBP.

#### 2.4.4 Statistical significance

All statistical tests were two-sided, and a *p*-value < 0.05 was considered statistically significant. Given the exploratory nature of some machine-learning analyses, no formal multiplicity correction was applied. Instead, emphasis was placed on effect sizes, confidence intervals, calibration performance, and consistency of findings across classical regression and machine-learning models. For prediction models, the primary emphasis was placed on out-of-sample discrimination (ROC-AUC and PR-AUC) and calibration (Brier score and calibration slope), rather than *p*-values.

## 3 Results

### 3.1 Sample characteristics by obesity phenotype and physical activity

A total of 2,191 community-dwelling older adults were included in the analytic sample following core variable filtering and complete-case screening. Based on the blood pressure-free obesity–metabolic phenotype definition (BMI plus lipid abnormalities only), participants were classified as MHNO (*n* = 1,003), MUNO (*n* = 889), MHO (*n* = 134), and MUO (*n* = 165) (Table 1A). Overall, the mean age ranged from 71.4 ± 4.8 years (MUO) to 73.1 ± 6.2 years (MHNO), and the proportion of women ranged from 46.3% (MHO) to 56.0% (MUNO). As expected, mean BMI was higher in obese phenotypes (MHO: 30.1 ± 2.4; MUO: 30.2 ± 1.9) than in non-obese phenotypes (MHNO: 23.4 ± 2.6; MUNO: 23.9 ± 2.5). Lipid profiles were consistent with phenotype definitions: TG was higher in metabolically unhealthy groups (MUNO and MUO: 2 [2, 3] mmol/L) than in metabolically healthy groups (MHNO: 1 [1, 1] mmol/L; MHO: 1 [1, 2] mmol/L), while HDL-C was lower in metabolically unhealthy groups (MUNO: 1.2 ± 0.3; MUO: 1.2 ± 0.2) than in metabolically healthy groups (MHNO and MHO: 1.5 ± 0.3). The median physical activity volume (PA Index, sessions × min) was 90 [0, 180] in MHNO and MUNO, and 180 [0, 180] in MHO and MUO (Table 1A).

Using the cohort-specific PA Index grouping, participants were categorised into inactive (*n* = 845), low active (*n* = 272), and high active (*n* = 1,074) (Table 1B). Participants in the inactive group were older (74.5 ± 6.9 years) than those in the low active (72.3 ± 5.4) and high active (71.4 ± 5.0) groups, while sex composition was comparable across PA strata (female: 50.8–52.2%). Hypertension prevalence increased from 67.3% in the inactive group to 80.1% in the low active and 81.7% in the high active groups (Table 1B). Specifically, the presence of all four phenotypes across the inactive, low active, and high active categories (Table 1C), including a substantial number of participants in the high active group within each phenotype, supports the separability of metabolic phenotype and activity volume and enables stable multivariable estimation of their independent associations with hypertension, as well as exploration of potential effect heterogeneity across metabolic risk strata.

Table 1C (phenotype × PA level) showed that MHNO (inactive/low active/high active: 386/132/485), MUNO (374/100/415), MHO (41/17/76), and MUO (44/23/98) were all represented across PA strata, indicating that activity volume was not concentrated within a single metabolic phenotype.

### 3.2 Prevalence of hypertension and cardiovascular outcomes

The overall prevalence of hypertension in the analytic sample was 75.9%. Across the blood pressure-free obesity–metabolic phenotypes, hypertension prevalence differed significantly, ranging from 72.9% in the MUNO group to 75.1% in MHNO, 85.1% in MHO, and 89.7% in MUO (Pearson’s *χ^2^* = 28.073, df = 3, *p* = 3.506 × 10^−6^) (Table 2; Figure 2). This gradient indicates heterogeneity in prevalent hypertension across BP-free phenotype strata, and the pattern persists despite blood pressure being strictly excluded from the phenotype definition.

ECG abnormalities were common across phenotypes, ranging from 54.7% (MUNO) to 64.8% (MUO), and did not differ significantly (Pearson’s *χ^2^* = 6.497, df = 3, *p* = 0.0898) (Table 2). No documented CHD history was recorded in this analytic dataset; therefore, CHD was described descriptively only and not modelled.

### 3.3 Collapsed obesity phenotype and cardiovascular outcomes

As a sensitivity and interpretability analysis, phenotypes were collapsed into two categories—non-obese (MHNO + MUNO) and obese (MHO + MUO)—to provide a policy-relevant contrast of adiposity status under the BP-free definition. In this binary comparison, obesity status was associated with a significantly higher likelihood of hypertension, corresponding to a 77% increase in odds (OR 1.77, 95% CI 1.21–2.58, *p* = 0.001). In contrast, obesity was not significantly associated with ECG abnormalities (OR 1.05, 95% CI 0.81–1.37, *p* = 0.76), suggesting no meaningful relationship between adiposity status and resting electrical cardiac abnormalities. This lack of association may be explained by the fact that ECG abnormalities are influenced by a complex array of factors beyond adiposity, such as chronic ageing processes and pre-existing comorbidities, which may dilute the direct impact of obesity status alone (Table 3).

### 3.4 Multivariable logistic regression

To address concerns regarding circular reasoning, multivariable logistic regression models were constructed using blood pressure-free obesity phenotypes and cohort-specific physical activity levels. In the fully adjusted model for hypertension (Table 4A), adiposity and physical activity were identified as independent risk factors after adjusting for age and sex.

Compared with the MHNO group, the MHO (OR 1.77, 95% CI 1.07–2.92, *p* = 0.026) and MUO groups (OR 2.65, 95% CI 1.56–4.49, *p* < 0.001) showed significantly higher odds of hypertension. Notably, a “physical activity paradox” was observed in this rural cohort: compared with inactive participants, those in the high active group exhibited a two-fold increase in hypertension risk (OR 2.00, 95% CI 1.61–2.49, *p* < 0.001). Additionally, older age was associated with slightly lower odds of hypertension (OR 0.98, *p* = 0.048), potentially suggesting a survivor bias.

For ECG abnormalities (Table 4B), older age remained the only significant independent predictor (OR 1.03, 95% CI 1.01–1.05, *p* = 0.009), while obesity phenotypes and physical activity levels did not show significant associations. In sensitivity analyses using a component-based specification (BMI, TG, and HDL-C instead of phenotype), the direction and statistical significance of the main associations were unchanged (data not shown).

### 3.5 Predictive performance of machine-learning models

To ensure pragmatic decision support in resource-limited rural settings where screening capacity may be intermittent (e.g., staffing and outreach logistics), we prioritised a “leakage-free” prediction framework. The primary LASSO logistic model was trained using only non-haemodynamic features (age, sex, BMI, lipids, and physical activity metrics).

**Figure 3:**
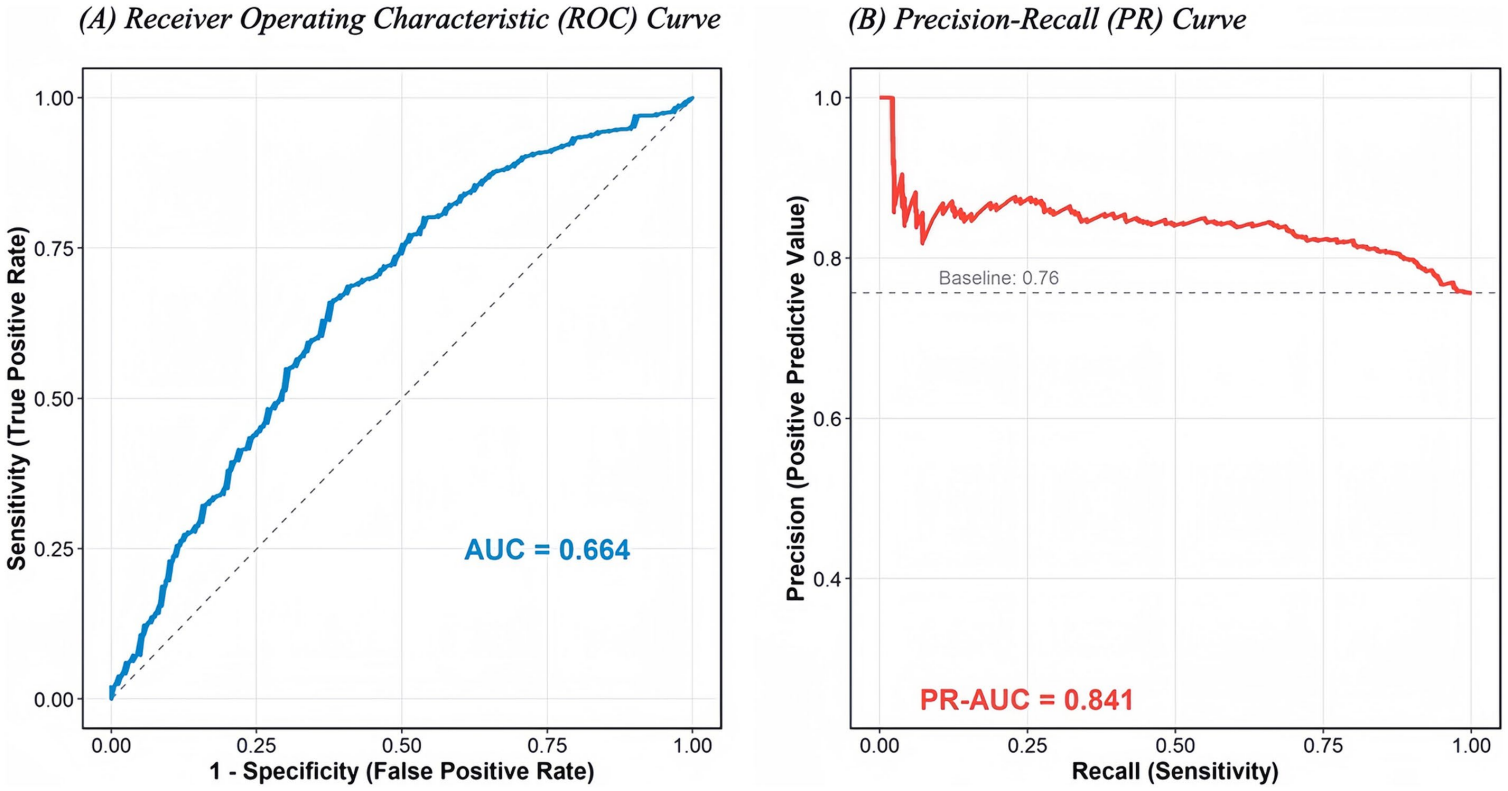
Discriminative performance of the leakage-free LASSO model for hypertension prediction. **(A)** Receiver Operating Characteristic (ROC) Curve. The area under the curve (AUC) is 0.664, indicating moderate discrimination using only non-haemodynamic predictors. **(B)** Precision–Recall (PR) Curve. The area under the PR curve (PR-AUC) is 0.841, demonstrating high precision in identifying hypertensive individuals in this high-prevalence cohort. The dashed line represents the baseline prevalence (0.76). *SBP and DBP were strictly excluded from the model predictors to avoid circular reasoning.*

On the held-out test set (*n* = 657), the leakage-free LASSO model demonstrated moderate discrimination with an AUC of 0.664 and a Precision–Recall AUC (PR-AUC) of 0.841 (Table 5; Figure 3). Given the high hypertension prevalence in this cohort (75.9%), the no-skill baseline for PR-AUC is approximately the prevalence (~0.76); therefore, PR-AUC = 0.841 should be interpreted as an incremental improvement beyond baseline in prioritising likely cases rather than high diagnostic performance. Calibration assessment indicated good agreement between predicted and observed probabilities, with a calibration slope of 1.10 and an intercept of −0.17 (Brier score: 0.173).

Decision threshold analysis was performed to facilitate triage-oriented implementation. Using the Youden-index-derived threshold (0.76), the model achieved a sensitivity of 66.0% and specificity of 62.5%. Furthermore, a sensitivity-priority threshold (0.66) yielded a sensitivity of 90.1% (Table 6), suggesting that the model may help prioritise follow-up and outreach scheduling in rural health programmes as a decision-support adjunct rather than a replacement for blood pressure measurement. Random Forest and XGBoost models yielded comparable AUCs (0.61–0.63) but did not surpass the parsimonious LASSO model in overall interpretability.

### 3.6 Feature importance and SHAP explainability

**Figure 4:**
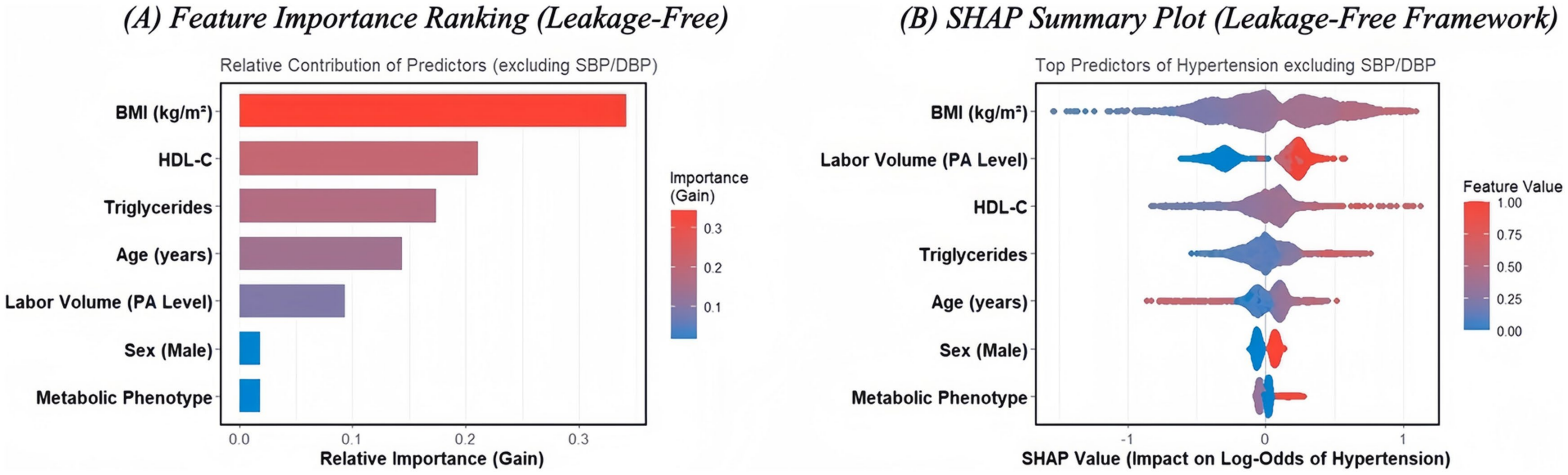
Explainability analysis of the leakage-free XGBoost model for hypertension prediction. **(A)** Feature Importance Ranking. Predictors are ranked by their contribution to the model’s discriminative performance (Gain). Adiposity (BMI) and physical activity volume are the top non-haemodynamic predictors. **(B)** SHAP Summary Plot. The beeswarm plot illustrates the direction and magnitude of feature impact on the log-odds of hypertension. Red points represent higher feature values. Notably, higher labour-type activity volume (high active status) is associated with positive SHAP values, indicating increased hypertension risk, consistent with the “physical activity paradox.” *Systolic and diastolic blood pressure were strictly excluded from the model to prevent circular reasoning.*

Permutation importance analysis from the leakage-free XGBoost model (excluding blood pressure) confirmed that obesity status (BMI) and physical activity volume (PA Index) were the dominant non-haemodynamic predictors of hypertension. Adiposity-related metrics resulted in the largest decreases in test-set AUC when permuted, followed by physical activity characteristics. In contrast, demographic factors such as age and sex showed relatively smaller contributions to the model’s discriminative performance in this specific rural cohort (Figure 4A).

SHAP summary analysis further elucidated these relationships at the individual level (Figure 4B). BMI and “high active” status exhibited the highest mean absolute SHAP values, identifying them as the primary drivers of the predicted hypertension probability. Higher BMI values were consistently associated with positive SHAP values, indicating increased log-odds of hypertension. Crucially, the SHAP analysis corroborated the “physical activity paradox”: high active status contributed positive SHAP values (represented by red points in the SHAP summary plot), shifting the model output toward a higher probability of hypertension. Triglycerides (TG) and HDL-C displayed moderate predictive influence, whereas smoking status and diet type had minimal impact on individual risk estimates.

Taken together, these explainability analyses underscore that adiposity and labour-type physical activity are the primary determinants of hypertension risk in this rural older population. These findings reinforce the clinical value of the leakage-free model, demonstrating that even without real-time blood pressure data, integrated metabolic and lifestyle profiling can effectively identify individuals at high risk for hypertension, providing a data-driven basis for prioritised screening.

## 4 Discussion

### 4.1 Public health significance: hypertension as a fundamental determinant of well-being in rural older adults

This study documents a substantial burden of hypertension among community-dwelling older adults in a cold-climate agricultural region. Using the blood pressure-free obesity–metabolic phenotypes, hypertension prevalence exhibited a significant gradient, increasing from 75.1% in the MHNO group to 89.7% in the MUO group (*χ^2^* = 28.07, *p* < 0.001). Hypertension is a well-established driver of cardiovascular morbidity, mortality, and accelerated biological ageing (31). Emerging evidence further indicates that elevated blood pressure undermines physical functioning, mobility confidence, social participation, independence, and subjective well-being—core pillars of successful and healthy ageing (32, 33). Accordingly, hypertension should be regarded not only as a biomedical condition but also as a fundamental determinant of late-life well-being and life satisfaction (1).

In rural China, structural health inequities magnify these consequences. Limited access to medical resources, delayed diagnosis, fragmented long-term management, and environmental constraints—especially in cold-climate agricultural settings—reduce opportunities for regular monitoring and prevention (34, 35). These vulnerabilities increase the likelihood of uncontrolled hypertension and accelerate transitions from physiological impairment to disability, dependence, and social withdrawal, threatening both physical autonomy and psychological security (5).

Despite the predominance of labour-type physical activity in this population, we observed widespread metabolic dysregulation, suggesting a mismatch between physical workload and cardiometabolic protection. Labour activity, characterised by repetitive mechanical tasks and irregular low-to-moderate intensity, may lack the sustained cardiovascular stimulus required to induce adaptive cardiorespiratory and vascular changes (36). Our findings, therefore, challenge the common assumption that “agricultural labour is equivalent to exercise,” and instead suggest that workload-driven physical strain can coexist with inadequate cardioprotective adaptation (37).

Taken together, these results highlight the importance of hypertension control as a central public health priority in rural ageing populations. Improving blood pressure is essential not only for preventing clinical events, but also for preserving functional independence, maintaining mobility, supporting social participation, and protecting holistic well-being among older adults in resource-constrained regions (6).

### 4.2 Physical activity, obesity phenotypes and cardiometabolic vulnerability: rethinking labour-based activity

Although labour-type activity was nearly universal in this cohort, guideline-based thresholds for structured exercise were not met, whereas cohort-specific PA Index grouping identified a large high-volume subgroup (high active, PA Index ≥ 180) likely reflecting agricultural labour exposure. This underscores a critical conceptual distinction: agricultural or domestic labour does not necessarily equate to structured exercise in terms of physiological stimulus or health impact (38). Labour activities often involve fluctuating intensity, repetitive mechanical loading, prolonged isometric exertion, and limited recovery, which may be accompanied by sustained sympathetic activation and vascular strain rather than the cardioprotective adaptations typically targeted by structured aerobic training (39, 40). The high prevalence of lipid abnormalities observed in this cohort is consistent with the possibility that “more work” does not inevitably translate into “better metabolic health” (41).

Across blood pressure-free obesity–metabolic phenotypes, hypertension prevalence ranged from 75.1% in MHNO to 89.7% in MUO, highlighting substantial cardiometabolic vulnerability beyond adiposity alone (42). The modest difference between MHNO (75.1%) and MUNO (72.9%) may reflect differences in age structure and residual confounding rather than a protective effect of lipid status, emphasising the need to interpret phenotype patterns alongside demographic and contextual factors (43).

A “physical activity paradox” pattern was observed in multivariable analyses: compared with inactive participants, the odds of prevalent hypertension were higher in the low active group (OR = 1.85, 95% CI 1.32–2.58) and the high active group (OR = 2.00, 95% CI 1.61–2.49; *p* < 0.001). While this contrasts with the common assumption that a higher workload is inherently protective, several alternative explanations should be considered. First, reverse causation is plausible: individuals aware of elevated blood pressure may have increased walking or reported more ≥30 min activity sessions following advice or health messaging. Second, the self-reported measure captures total volume but lacks information on intensity, recovery, and seasonality, and it mixes labour and walking; therefore, the observed association should be interpreted as relating to reported workload context rather than implying a harmful causal effect of physical activity per se. Third, residual confounding (e.g., health consciousness, care-seeking, occupational role, and unmeasured lifestyle factors) may influence both reported activity patterns and hypertension detection. In Northeast China, high-volume labour frequently occurs under cold stress and may involve heavy lifting and static posturing, which can acutely elevate blood pressure through peripheral vasoconstriction; this provides a biologically plausible context for co-occurrence, but longitudinal evidence is required to clarify temporal pathways (44, 45).

Collectively, these cross-sectional findings highlight the need to reconsider community beliefs about physical activity in labour-dominant rural settings. Rather than equating physical fatigue with healthfulness, community programmes may consider incorporating evidence-informed, structured exercise options—such as supervised, progressively dosed aerobic and resistance training—aimed at supporting cardiopulmonary fitness, metabolic flexibility, and functional independence in older adults, while recognising that effectiveness and feasibility warrant evaluation in longitudinal and implementation studies (46).

### 4.3 Innovation strategies: AI-enabled leakage-free machine learning for targeted hypertension screening

Methodologically, this study developed an AI-enabled prediction framework that excludes systolic and diastolic blood pressure from model inputs to reduce circularity and avoid performance inflation from outcome-defining variables (47). Under this conservative specification, the leakage-free LASSO model achieved a ROC-AUC of 0.664 and a PR-AUC of 0.841 on the held-out test set (*n* = 657), with good calibration (slope = 1.10; intercept = −0.17; Brier score = 0.173). Because hypertension prevalence was high in this cohort, PR-AUC should be interpreted relative to the baseline prevalence (~0.76), and the observed PR-AUC (0.841) indicates incremental improvement for case prioritisation rather than high diagnostic performance. The model used routinely collected variables (e.g., age, sex, BMI, triglycerides, HDL-C, and PA metrics), suggesting potential utility as a decision-support adjunct for programme planning.

From an implementation perspective, structural health inequalities and resource constraints are central considerations in rural China (48). This framework is not intended to replace blood pressure measurement—an essential and low-cost clinical assessment—but rather to support prioritised follow-up when screening capacity is intermittent (e.g., staffing, outreach logistics, seasonal constraints, or competing service demands). A pragmatic deployment pathway could operate with minimal technological burden at the village level: county-level systems can generate prioritised follow-up lists from existing examination records, while township/village teams use these lists to schedule repeat measurements, outreach visits, and health education. Such a workflow aligns with the reality that technological resources and trained personnel are unevenly distributed and that digital tools must be lightweight, interpretable, and integrated into existing public health routines (49).

Interpretability remains important for adoption. Explainability analyses (permutation importance and SHAP) indicated that BMI and labour-type activity volume (PA Index/high active status) contributed substantially to predicted probabilities, with lipid markers providing additional information. The positive SHAP contribution of high active status is consistent with the observed epidemiological association and helps clarify how workload-related features influence model output, although it does not establish causality (50). In combination, these features suggest potential advantages of an AI-assisted approach when used cautiously: (1) risk stratification to support outreach and follow-up scheduling; (2) support for tailored, context-adapted lifestyle guidance; and (3) a scaffold for chronic disease management workflows that link screening, follow-up, and feedback. These directions align with national initiatives promoting active ageing, “Internet+ healthcare,” and sports–medicine integration, but require implementation evaluation to confirm feasibility, acceptability, and cost-effectiveness under real-world inequality constraints (51).

### 4.4 Policy and practical implications: building a community-based, activity-centred model for healthy ageing

Our findings provide actionable guidance for designing community-centred strategies to protect cardiovascular health and enhance well-being among rural older adults. In the context of rapidly accelerating population ageing and limited health-service capacity in rural regions, reactive treatment alone is insufficient to control the rising hypertension burden and prevent functional decline (52). In this cross-sectional analysis, the observed associations are consistent with the possibility that labour-based physical activity may not replicate the cardioprotective stimulus of structured exercise and that higher reported activity volume could co-occur with greater cardiovascular strain in contexts where intensity, recovery, and environmental stressors are not well managed. These findings also suggest that BP-free metabolic risk stratification, together with structured exercise programmes, may warrant consideration as part of community prevention efforts, pending confirmation in longitudinal and intervention studies.

To address these challenges, we propose a community-based, AI-supported pre-measurement triage and closed-loop model that integrates public health surveillance, precision exercise prescription, and intelligent chronic disease management (53):

1. Strengthened cardiovascular monitoring.
2. Regular blood pressure assessment, lipid testing, and personalised feedback delivered through village clinics to improve early detection and reduce undiagnosed hypertension.
3. Sports science-informed exercise prescription.
4. Design of individualised programmes that complement labour demands through appropriately dosed aerobic and resistance training, targeting endothelial function, vascular elasticity, and autonomic balance (54).
5. AI-supported risk prediction and decision support.
6. Deployment of explainable machine-learning models to assist village doctors and community health workers in prioritising high-risk individuals, guiding follow-up scheduling, and tailoring intervention intensity.
7. Activity-supportive rural environments.
8. Construction of age-friendly walking infrastructure, indoor winter exercise spaces, and community activity hubs to overcome extreme cold seasonal constraints characteristic of Northeast China (55).

From a policy perspective, this proposed model aligns with Healthy China 2030, Healthy Heilongjiang, and the national strategy of integration of sports and medicine. Strengthening collaboration among public health administrations, primary care institutions, sports science professionals, and digital health platforms may shift chronic disease management from episodic clinical responses toward a dynamic, lifelong prevention system, thereby improving physical independence, reducing psychological burden, enabling social participation, and promoting dignity in later life (56).

**Figure 5:**
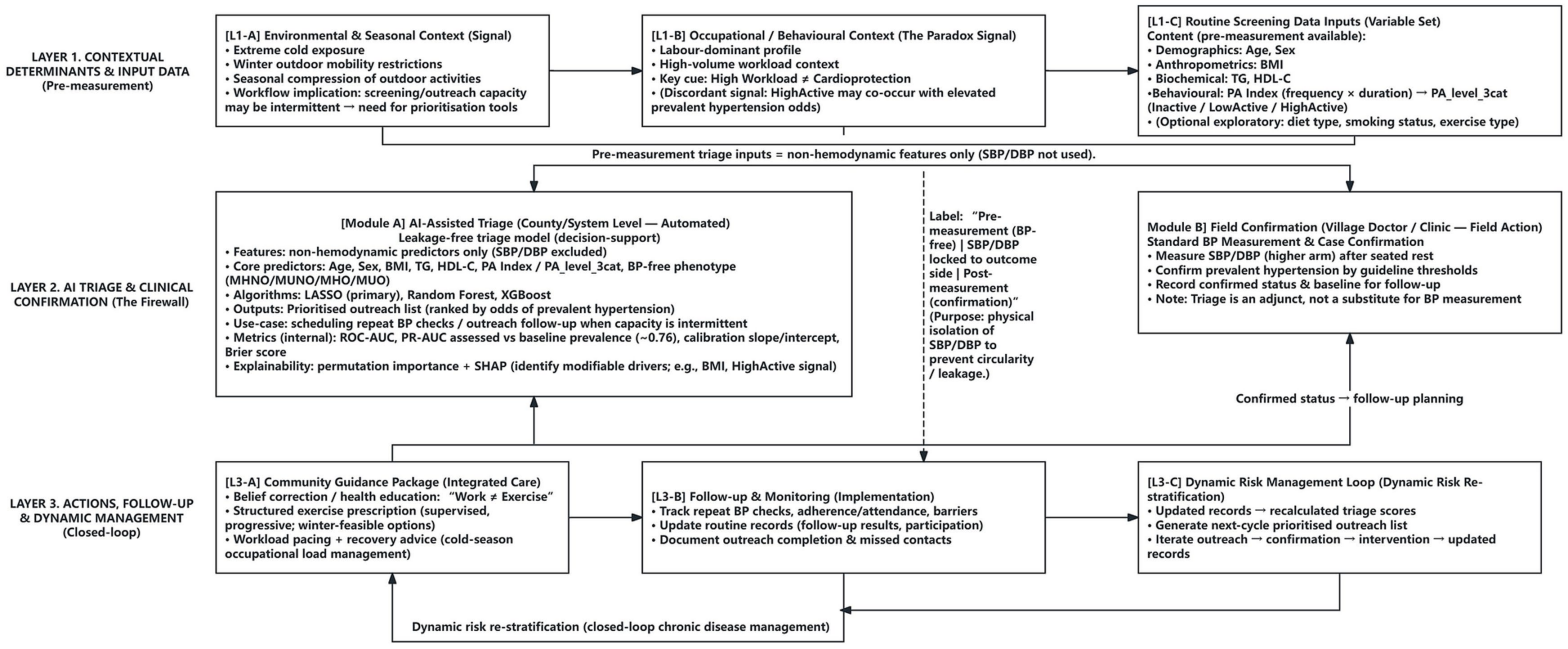
AI-supported leakage-free pre-measurement triage, field confirmation, and closed-loop follow-up workflow for prevalent hypertension in cold-climate rural ageing populations (decision-support adjunct; not a substitute for blood pressure measurement).

A conceptual workflow for this community-based, AI-supported “pre-measurement triage → field confirmation → follow-up → dynamic re-stratification” approach is illustrated in Figure 5. The figure intentionally presents an implementation-oriented decision-support pathway rather than a causal mechanism model: (i) non-haemodynamic routine screening inputs (demographics, BMI, lipids, and PA metrics) are used at the county/system level to generate a prioritised outreach list under a leakage-free “firewall” (SBP/DBP excluded); (ii) village doctors or clinics perform standardised blood pressure measurement to confirm prevalent hypertension; and (iii) confirmed status triggers follow-up planning, context-adapted lifestyle/exercise guidance, and record updating to enable dynamic risk re-stratification. This framing is consistent with the cross-sectional nature of the evidence and positions the proposed strategy as a pragmatic adjunct to support outreach scheduling and resource prioritisation in cold-climate rural settings, pending longitudinal validation and implementation evaluation.

### 4.5 Limitations and future directions

First, the cross-sectional design precludes causal inference. All findings should be interpreted as associations rather than effects, and reverse causation remains plausible (e.g., individuals with known or suspected hypertension may have modified their activity patterns or care-seeking behaviours). Although the analytic sample increased to *N* = 2,191—improving the stability of the MHO subgroup (*n* = 134)—temporality cannot be established, and unmeasured factors (e.g., frailty, health consciousness, occupational role, or access to care) may influence both reported activity and hypertension detection. Longitudinal cohorts and intervention studies are therefore required to clarify temporal pathways and to evaluate whether structured exercise programmes improve blood pressure profiles in rural ageing populations (57).

Second, physical activity was self-reported and operationalised as weekly sessions of ≥30 min (including labour and walking), which may introduce recall bias and exposure misclassification. The cohort-specific PA Index captures volume but cannot separate labour-related mechanical strain from leisure-time aerobic activity, nor quantify intensity, recovery, posture (e.g., isometric work), or seasonality—factors that may be particularly relevant in cold-climate agricultural contexts. Consequently, the observed “physical activity paradox” should be interpreted as reflecting a workload-related exposure context rather than implying that physical activity is harmful. Future studies should incorporate objective measures (e.g., accelerometry, GPS, and heart-rate monitoring) and training-load metrics (intensity × duration × frequency), enabling a clearer distinction between labour and structured exercise and more robust dose–response assessment (58).

Third, confounding control was limited by the scope of routinely collected examination variables. Key determinants such as socioeconomic status/education, detailed diet (especially sodium intake), alcohol use, medication adherence, psychosocial stress, sleep, functional status, and healthcare accessibility were unavailable or crudely captured, which may bias estimated associations. Incorporating these variables—along with environmental indicators (e.g., cold exposure, heating conditions, walkability)—could strengthen both aetiologic interpretation and pragmatic intervention design (59).

Fourth, outcome ascertainment from routine records has inherent constraints. While blood pressure measurement was standardised, single-occasion readings may be influenced by short-term variability. In addition, no documented CHD history was recorded in the analytic dataset; this likely reflects underdiagnosis and/or recording limitations in routine village-level health examination forms (e.g., reliance on previously documented physician diagnoses rather than systematic adjudication), rather than true absence of CHD in this population. Accordingly, CHD was reported descriptively only and not modelled.

Fifth, the machine-learning component was intentionally specified as leakage-free (SBP/DBP excluded), which improves methodological integrity but also limits discrimination. Model performance should therefore be interpreted as incremental case-prioritisation utility, not diagnostic replacement of blood pressure measurement. External validation was not available; thus, performance estimates may not generalise to other regions, seasons, or service settings. Future work should evaluate external transportability and implementation feasibility, including cost and workflow integration under rural inequality constraints (60).

Finally, the sample was drawn from a single cold-climate agricultural county, limiting generalisability to settings with different occupational structures, cultural norms, and healthcare access. Multi-site rural cohorts and cross-provincial validation studies are needed to test robustness and support translation (61).

#### 4.5.1 Future research roadmap

To strengthen evidence and accelerate translation, we propose:

1. Longitudinal follow-up using repeated annual examinations to test the temporal stability of the “work–exercise paradox.”
2. Pragmatic community trials comparing structured, progressively dosed exercise programmes with usual labour-dominant activity contexts.
3. Integration of wearable-derived training-load and cold-season exposure indicators to refine dose–response inference.
4. Multi-centre rural studies across diverse climatic and socioeconomic contexts.
5. Implementation evaluation of an AI-supported triage workflow embedded within existing primary-care outreach systems (effectiveness, acceptability, and cost-effectiveness).

Building on the next annual examination cycle, we plan a 2026 extension to establish a longitudinal cohort, enabling temporal testing of observed associations, improved CHD ascertainment via linkage with clinic/hospital records where feasible, and incorporation of objective activity and cold-season exposure metrics to strengthen inference and implementation relevance.

### 4.6 Key message

Improving cardiovascular health is a foundational pathway to sustaining well-being, autonomy, and functional independence among rural older adults. In this cross-sectional cohort, higher labour-type activity volume was independently associated with higher odds of prevalent hypertension (high active vs. inactive: OR = 2.00, 95% CI 1.61–2.49), a pattern consistent with a “physical activity paradox” in a cold-climate agricultural context. Using blood pressure-free obesity–metabolic phenotypes, hypertension prevalence varied across groups (MHNO 75.1% to MUO 89.7%), indicating heterogeneity in cardiometabolic vulnerability beyond adiposity alone. Taken together, these findings do not support a simple assumption that “work equals exercise” and instead suggest that labour-related workload may not replicate the cardioprotective stimulus of structured exercise; however, causal interpretations are not warranted without longitudinal and intervention evidence.

By combining metabolic phenotype stratification with AI-enabled, leakage-free prediction (ROC-AUC 0.664; PR-AUC 0.841; calibration slope 1.10), we outline a pragmatic decision-support approach to help prioritise follow-up and outreach when screening capacity is intermittent in resource-limited rural settings. Given the high baseline prevalence of hypertension, this prediction framework should be viewed as providing incremental value for case prioritisation and programme planning, and it is intended to assist rather than replace standard blood pressure measurement. Together, these results support further evaluation of integrated strategies that link metabolic risk profiling, context-adapted activity guidance, and scalable follow-up to reduce rural–urban health inequities and promote active, dignified ageing.

## 5 Conclusion

This cross-sectional study in a cold-climate agricultural community identified a substantial hypertension burden among rural older adults. Using blood pressure-free obesity–metabolic phenotypes (defined by BMI and lipid profiles only), we observed a graded pattern across phenotypes, indicating that metabolic dysregulation was associated with hypertension in this setting, beyond obesity status alone. Although labour-type activity was nearly universal, guideline-based “high activity” thresholds were not met, and our analysis using cohort-specific groupings suggested that higher self-reported activity volume was associated with increased hypertension odds. This pattern is consistent with a potential “physical activity paradox” in non-volitional rural labour contexts, possibly reflecting occupational strain, cold exposure, and cumulative workload rather than cardioprotective exercise stimuli. However, these results should be interpreted cautiously; due to the cross-sectional design, they do not establish causal effects and may be influenced by reverse causation or unmeasured confounding.

By integrating classical epidemiological analyses with a leakage-free machine learning framework, we demonstrate that routinely collected screening data can serve as a complementary decision-support tool to prioritise follow-up, outreach, or repeated blood pressure measurements when screening capacity is limited or intermittent. Importantly, this approach is intended to assist, not replace, formal blood pressure assessment in resource-limited rural settings.

Overall, our results highlight the critical intersection of ageing, labour-type work, and cardiovascular health. They underscore the need to move beyond the belief that “work equals exercise” toward an evidence-based paradigm that combines metabolic risk stratification and pragmatic, explainable AI-supported triage. Future longitudinal research is warranted to verify whether structured and progressively dosed exercise can mitigate the cardiovascular risks associated with heavy rural labour in cold-climate regions.

## Data Availability

The raw data supporting the conclusions of this article will be made available by the authors, without undue reservation.
